# Exposure to Multiple Parasites Is Associated with the Prevalence of Active Convulsive Epilepsy in Sub-Saharan Africa

**DOI:** 10.1371/journal.pntd.0002908

**Published:** 2014-05-29

**Authors:** Gathoni Kamuyu, Christian Bottomley, James Mageto, Brett Lowe, Patricia P. Wilkins, John C. Noh, Thomas B. Nutman, Anthony K. Ngugi, Rachael Odhiambo, Ryan G. Wagner, Angelina Kakooza-Mwesige, Seth Owusu-Agyei, Kenneth Ae-Ngibise, Honorati Masanja, Faith H. A. Osier, Peter Odermatt, Charles R. Newton, on behalf of the Study of Epidemiology of Epilepsy in Demographic Sites (SEEDS) group

**Affiliations:** 1 KEMRI/Wellcome Trust Research Programme, The Centre of Geographical Medicine Research – Coast, Kilifi, Kenya; 2 Studies of the Epidemiology of Epilepsy in Demographic Surveillance Systems (SEEDS)-INDEPTH Network, Accra, Ghana; 3 Department of Infectious Disease Epidemiology, Faculty of Epidemiology and Population Health, London School of Hygiene and Tropical Medicine, London, United Kingdom; 4 MRC Tropical Epidemiology Group, Faculty of Epidemiology and Population Health, London School of Hygiene and Tropical Medicine, London, United Kingdom; 5 Faculty of Infectious and Tropical Diseases, London School of Hygiene and Tropical Medicine, London, United Kingdom; 6 Egerton University, Nakuru, Kenya; 7 Nuffield Department of Clinical Medicine, University of Oxford, Oxford, United Kingdom; 8 Division of Parasitic Diseases and Malaria, Centers for Disease Control and Prevention (CDC), Atlanta, Georgia, United States of America; 9 Laboratory of Parasitic Diseases. National Institute of Allergy and Infectious Diseases, Bethesda, Maryland, United States of America; 10 Research Support Unit, Faculty of Health Sciences, Aga Khan University (East Africa), Nairobi, Kenya; 11 MRC/Wits Rural Public Health & Health Transitions Research Unit (Agincourt), School of Public Health, Faculty of Health Sciences, University of the Witwatersrand, Johannesburg, South Africa; 12 Epidemiology and Public Health, Department of Public Health and Clinical Medicine, Umeå University, Umeå, Sweden; 13 Iganga-Mayuge Health and Demographic Surveillance System, Iganga, Uganda; 14 Department of Paediatrics and Child Health, Makerere University College of Health Sciences, Kampala, Uganda; 15 Kintampo Health Research Centre, Kintampo, Ghana; 16 Ifakara Health Institute, Ifakara, Tanzania; 17 Swiss Tropical and Public Health Institute, Basel, Switzerland; 18 Unversity of Basel, Basel, Switzerland; 19 Neurosciences Unit, UCL Institute of Child Health, London, United Kingdom; 20 Clinical Research Unit, London School of Hygiene and Tropical Medicine, London, United Kingdom; 21 Department of Psychiatry, University of Oxford, Oxford, United Kingdom; Universidad Peruana Cayetano Heredia, Peru

## Abstract

**Background:**

Epilepsy is common in developing countries, and it is often associated with parasitic infections. We investigated the relationship between exposure to parasitic infections, particularly multiple infections and active convulsive epilepsy (ACE), in five sites across sub-Saharan Africa.

**Methods and Findings:**

A case-control design that matched on age and location was used. Blood samples were collected from 986 prevalent cases and 1,313 age-matched community controls and tested for presence of antibodies to *Onchocerca volvulus*, *Toxocara canis*, *Toxoplasma gondii*, *Plasmodium falciparum*, *Taenia solium* and HIV. Exposure (seropositivity) to *Onchocerca volvulus* (OR = 1.98; 95%CI: 1.52–2.58, p<0.001), *Toxocara canis* (OR = 1.52; 95%CI: 1.23–1.87, p<0.001), *Toxoplasma gondii* (OR = 1.28; 95%CI: 1.04–1.56, p = 0.018) and higher antibody levels (top tertile) to *Toxocara canis* (OR = 1.70; 95%CI: 1.30–2.24, p<0.001) were associated with an increased prevalence of ACE. Exposure to multiple infections was common (73.8% of cases and 65.5% of controls had been exposed to two or more infections), and for *T. gondii* and *O. volvulus* co-infection, their combined effect on the prevalence of ACE, as determined by the relative excess risk due to interaction (RERI), was more than additive (*T. gondii* and *O. volvulus*, RERI = 1.19). The prevalence of *T. solium* antibodies was low (2.8% of cases and 2.2% of controls) and was not associated with ACE in the study areas.

**Conclusion:**

This study investigates how the degree of exposure to parasites and multiple parasitic infections are associated with ACE and may explain conflicting results obtained when only seropositivity is considered. The findings from this study should be further validated.

## Author Summary

The prevalence of epilepsy is greater in developing countries compared to developed countries, and parasitic infestations are thought to contribute to this increased burden. We conducted a case-control study across five sites in sub-Saharan Africa to investigate the relationship between epilepsy and exposure to parasitic infections, and the association between epilepsy and multiple co-incidental infections. Exposure to *Onchocerca volvulus, Toxocara canis* and *Toxoplasma gondii* as well as high antibody levels (top tertile) to *Toxocara canis* was positively associated with the prevalence of active convulsive epilepsy (ACE). Multiple co-incidental parasitic infections were common, and the combined effect of *T. gondii* and *O. volvulus* co-infection on ACE was greater than the sum of the individual effects. The contribution of each of these parasitic infections on the burden of epilepsy in sub-Saharan Africa should be explored.

## Introduction

The prevalence of epilepsy in low and middle-income countries is higher than in high-income countries, especially in the rural areas[1], [2]. The prevalence is particularly high in sub-Saharan Africa (SSA)[3] and South America[4], where parasitic infestations are thought to contribute to the increased burden[5]. Within these regions, there are areas in which most of the population are exposed to endemic parasites, and it is not clear why some people develop epilepsy, whilst others do not.

Many factors are associated with epilepsy in SSA[3], [6] with infections that involve the central nervous system (CNS) representing common and preventable causes of epilepsy[5]. Some parasitic infestations manifest in the human CNS, with the clinical presentation of seizures and are thought to be associated with the development of epilepsy[5], [7]. A small number of studies conducted in SSA have shown that exposure to helminths, e.g., *Toxocara canis* [8], [9], [10], *Onchocerca volvulus* [10], [11], [12], [13] and *Taenia solium* [10], [14], as well as following severe *Plasmodium falciparum* malaria[15],[16] are associated with epilepsy. The relationship between *Toxoplasma gondii* and epilepsy has only been explored in one study in SSA[10], and a review suggests a possible association[17], though co-infection with human immunodeficiency virus may confound this relationship. Seizures are observed in HIV-infected individuals and are mainly associated with opportunistic infections although HIV infection can independently cause seizures at seroconversion or at advanced stages[18]. A comprehensive analysis of exposure to parasitic infestations as well as HIV using the same methodology across different geographical locations in SSA would help elucidate the relationship between parasitic infections and epilepsy, and provide data to guide public health measures.

The objective of the current study was to investigate the association between active convulsive epilepsy (ACE) and i) the degree of exposure to parasitic infections (measured by antibody levels) and ii) exposure to multiple co-incidental parasitic infections. We used data from a case-control study conducted in five health and demographic surveillance systems (HDSS) in SSA in which exposure to the six infections namely: *O. volvulus*, *T. solium*, *T. canis*, *T. gondii*, *P. falciparum* and HIV, was determined by serology.

## Methods

### Ethics statement

All aspects of the study were approved by the ethics committees of University College London and the London School of Hygiene and Tropical Medicine, and by the ethics review boards in each of the participating countries. All participants or guardians gave written informed consent. Since some study participants were minors, parents/guardians provided consent on behalf of all child participants and all adults provided consent for themselves.

### Participants and study design

A case control design was used in which prevalent cases of ACE were compared to community controls. ACE was defined as two or more unprovoked convulsions (seizures with tonic and/or clonic movements) occurring at least 24 hours apart with at least one seizure in the preceding 12 months[6]. The diagnosis for ACE was initially made by a clinician with special training in epilepsy, and was confirmed by a panel of neurologists.

Cases and controls were identified from five HDSS in SSA (Agincourt in South Africa, Kilifi in Kenya, Kintampo in Ghana, Ifakara in Tanzania and Iganga-Mayuge in Uganda). Exposure to parasitic infections was measured using plasma samples collected from a random subsample of all cases and controls. A sample size of 300 cases per site with an equal number of controls was chosen to give 80% power to detect an odds ratio (OR) >2.8 (5% significance level) given a frequency of at least 5% in the controls. For each case, an age-matched control was selected at random from a database of individuals in the HDSS. The controls were frequency matched using the age bands 0–5, 6–12, 13–18, 19–28, 29–49, 50+ years, to account for increasing exposure with age.

### Laboratory procedures

Exposure to infections was determined by detection of IgG antibodies to the parasitic antigens as well as HIV. Exposure to *O. volvulus* and *T. solium* was determined in three study sites: Iganga, Ifakara and Kintampo, where these parasites are endemic.

#### Exposure to *Onchocerca volvulus*

Exposure to *O. volvulus* was determined using an in-house modification of an anti-OV-16GST IgG4 ELISA described previously[19]. This assay is highly sensitive (90.0%) and specific (98.0%) when tested using sera from other filarial infections[19]. A sample with an optical density (OD) value greater than the cut-off (mean +3 standard deviation of 30 plasma samples from Agincourt, South Africa, in which onchocerciasis is absent) was classified as being anti-OV16gst IgG4 positive.

#### Exposure to larval and adult stages of *Taenia solium*

Exposure to larval (cysticercosis) and adults (taeniasis) stages of *T. solium* was determined using a Western blotting technique that has been described previously[20]. Nitrocellulose strips that contained the recombinant rT24H and rES33 antigens, which detect antibodies to cysticercosis and taeniasis respectively, were used. Samples were considered positive for cysticercosis or taeniasis if a brown band was observed in regions of the strip corresponding to rT24H or rES33 antigen respectively[21], [22]. A similar technique (MAPIA), is reported to be 97.0% sensitive and 99.4% specific for detection of cases with two or more viable cysts in the brain and 99.4% sensitive and 93.9% specific for detecting cases of taeniasis[20].

#### Exposure to *Toxocara canis*

Anti-*T. canis* total IgG antibodies were detected using a commercial kit (*Toxocara* IgG-ELISA, Cypress Diagnostics, Belgium) that is based on the Toxocara excretory secretory antigen (TES) and used according to manufacturer's instructions. The cut-off value was calculated by dividing the sample test OD by the average OD of negative controls plus 0.150 OD units according to manufacturer's instructions. A sample with a cut-off ratio >1.1 was interpreted as positive and tested for anti-*Toxocara* IgG4 antibodies, to increase the specificity of the assay. Anti-*Toxocara* IgG4 antibodies were detected using the same TES-precoated plates with the following modifications. Mouse anti-human IgG4 conjugated to alkaline phosphatase (9190–04, Southern Biotech, USA) as the secondary antibody and p-nitrophenyl phospate (pNPP) (N2765, Sigma Aldrich) as the substrate. Serum dilution (1∶50) and secondary antibody dilution (1∶500) was optimized by checkerboard titration. A sample with an OD value greater than the cut-off (mean +3 standard deviation of 30 *Toxocara canis* IgG negative serum samples) was interpreted as being anti-*Toxocara* IgG4 positive. The *T. canis* assay is reported to be 97.0% sensitive and 78.6% specific[23] and cross-reactive responses from other soil-transmitted helminths may have been detected using this assay.

#### Exposure to *Toxoplasma gondii*

Anti-*T. gondii* IgG antibodies were detected using a commercial kit (*Toxoplasma* IgG-ELISA, Genesis Diagnostics, United Kingdom) that is based on *T. gondii* purified antigens enriched for P30 (SAG1) and used according to manufacturer's instructions.

#### Exposure to *Plasmodium falciparum*

Exposure to *P. falciparum* was determined using an in-house ELISA described previously[24] that tests for IgG antibodies to crude schizont extract from *P. falciparum* A4 clone line derived from ITO parent strain. A sample with an OD value greater than the cut-off (mean +3 standard deviation of 30 serum samples from unexposed adults from United Kingdom) was interpreted as being anti-*P. falciparum* IgG positive.

#### Exposure to HIV

Anti- HIV-1 type and/or anti-HIV-2 type IgG antibodies and P24 antigen were detected by the 4^th^ generation screening test, Vironostika HIV Uniform II Ag/Ab (BioMerieux, France) and was performed according to manufacturer's instructions. The HIV assay was selected as it detects antibodies to both HIV-1 and HIV-2 type strains whose geographic distribution varies across SSA.

### Statistical analysis

Logistic regression was used to model the association between antibody titre and ACE.

Antibody titre was categorised into tertiles, which were calculated separately for each study site. The model also included, as potential confounders: age (0–5, 6–12, 13–18, 19–28, 29–49, 50+ years), sex, study-site, education (none, primary, or secondary and above), employment and marital status.

Logistic regression was also used to model exposure to multiple infections. We used adjusted odds ratios obtained from logistic regression to test for an additive interaction between parasites by calculating the relative excess risk due to interaction (RERI). The analysis of interactions was restricted to those parasites where the association with epilepsy was statistically significant (p<0.05). We briefly outline the relation between RERI and interaction on an additive scale.

Under an additive model the following relation holds
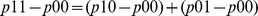
where 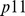 and 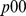 correspond to the prevalence when both or neither parasite risk factor is present, and 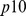 and 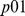 represent the prevalence when one of the risk factors is present. An interaction on an additive scale can be quantified as the difference between 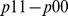 and 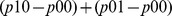. If this difference is positive then the combined effect of the two parasites is more than the sum of their individual effects. Conversely, if the difference is negative then the combined effect is less than the sum of the individual effects. The RERI is obtained by dividing the difference by 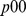




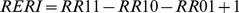



Again a positive RERI represents a combined effect that is greater than that predicted by the additive model and a negative RERI occurs when the combined effect that is less than additive. We use odds ratios obtained from logistic regression to approximate prevalence ratios in the above formula; this approximation is expected to work well since epilepsy is rare in the study population[25]. No adjustment for multiple comparisons was made because this adjustment can lead to errors of interpretation when strong previous evidence of association is available for several risk factors [26]. All analyses were conducted using STATA version 12 (StataCorp.College Station, TX, USA).

## Results

### Antibody prevalence and prevalence of ACE

2,032 controls and 1,711 cases were recruited. The sensitivity of the three-stage survey method was 48.6%[27], and among controls the rate of refusal ranged between 49.8% in Agincourt to 57.4% in Kilifi[10]. Blood samples were collected from 986 cases of ACE and 1,313 controls. The number of blood samples tested for each parasite were as follows: *O. volvulus*, 535 cases and 836 controls; *T. canis*, 862 cases and 1,121 controls; *T. gondii* 971 cases and 1,291 controls; *P. falciparum*, 986 cases and 1,313 controls; HIV, 977 cases and 1,304 controls; *T.solium*, 530 cases and 833 controls. The demographic details of the cases and controls from each study site are shown in Table S1.

The prevalence of antibodies to the six infections studied varied between the five study sites (Table 1). The prevalence of antibodies increased with age in both cases and controls for *O. volvulus, T. canis, T. gondii* and *P. falciparum* (Figure S1–S3, respectively) and these trends were consistent in all study sites. Almost all study participants were *P. falciparum* positive in three sites (Figure S4). Antibody prevalence to *T. solium* was low in the three study sites analysed (Table 1) and showed no trend with age (Figure S5). Similarly, antibody prevalence to HIV showed no trend with age (Figure S6).

**Table 1:** Table showing the seropositivity to six different infections and association with ACE in the five study sites.

	All study sites			Agincourt, South Africa			Ifakara, Tanzania			Iganga-Mayuge, Uganda			Kilifi, Kenya			Kintampo, Ghana		
	Control%	Case%	OR*	Control%	Case%	OR**	Control%	Case%	OR**	Control%	Case%	OR**	Control%	Case%	OR**	Control%	Case.%	OR**
			(95% CI)			(95% CI)			(95% CI)			(95% CI)			(95% CI)			(95% CI)
			P-value			P-value			P-value			P-value			P-value			P-value
No of Individuals	1313	986		211	175		345	278		199	84		266	276		292	173	
*Plasmodium falciparum* +	82.8	81.7	1.12	33.2	40.0	1.34	94.2	97.1	1.96	99.5	100.0	-	76.3	75.7	0.88	99.7	100.0	-
			(0.84–1.50)			(0.84–2.13)			(0.81–4.78)						**(0.54–1.41)**			
			0.445			0.219			0.138						**0.587**			
HIV +	14.0	14.3	1.07	23.6	18.9	0.79	12.8	16.2	1.26	2.5	2.4	1.70	2.3	5.9	**3.0**	26.7	25.4	0.81
			(0.82–1.39)			(0.45–1.39)			(0.78–2.04)			(0.27–10.83)			**(1.12–8.04)**			(0.51–1.31)
			0.622			0.409			0.340			0.574			**0.028**			0.402
*Toxocara canis* +	22.3	31.2	**1.52**	11.9	10.3	0.92	26.3	43.5	**2.15**	24.6	22.6	0.84	29.3	41.7	1.64	17.8	21.9	1.30
			**(1.23–1.87)**			(0.43–1.91)			**(1.46–3.18)**			(0.41–1.71)			(1.11–2.40)			(0.79–2.15)
			**<0.001**			0.817			**<0.001**			0.632			0.012			0.301
*Toxoplasma gondii* +	35.4	39.1	**1.28**	9.9	12.0	1.29	42.4	48.7	1.18	27.6	25.0	0.97	28.6	31.9	1.34	57.5	70.5	1.55
			**(1.04–1.56)**			(0.63–2.64)			(0.83–1.70)			(0.49–1.91)			(0.88–2.02)			(0.98–2.44)
			**0.018**			0.480			0.349			0.922			0.168			0.059
*Onchocerca volvulus +*	22.6	37.8	**1.98**	na	na	na	29.6	40.3	**1.52**	6.0	11.9	**2.89**	na	na	na	25.7	46.2	**2.59**
			**(1.52–2.58)**						**(1.06–2.19)**			**(1.08–7.69)**						**(1.66–4.04)**
			**<0.001**						**0.023**			**0.034**						**<0.001**
Cysticercosis +	1.1	1.8	1.85	na	na	na	0.3	1.8	-	0.0	2.4	-	na	na	na	2.8	1.7	-
			(0.73–4.70)															
			0.195															
Taeniasis +	1.2	1.3	1.42	na	na	na	0.0	1.5	-	1.0	0.0	-	na	na	na	2.7	1.7	-
			(0.51–4.00)															
			0.503															
Cysticercosis and Taeniasis+	2.2	2.8	1.54	na	na	na	0.3	2.9	15.42	1.0	2.4	4.16	na	na	na	5.2	2.9	0.58
			(0.74–3.20)						**(1.84–129.1)**			(0.26–65.65)						(0.20–1.71)
			0.245						**0.012**			0.311						0.322

*Odds ratio (OR) adjusted for age, sex, study site, education (none, primary, or secondary and above), employment and marital status.

**Odds ratio (OR) adjusted for age, sex, education (none, primary, or secondary and above), employment and marital status. na- not applicable.

The association between seropositivity and ACE, varied between different exposures and study sites (Table 1). Significant associations with ACE include exposure to *O. volvulus* in all study sites (OR = 1.98; 95%CI: 1.52–2.58, p<0.001), Ifakara (OR = 1.52; 95%CI: 1.06–2.19, p = 0.023), Iganga-Mayuge (OR = 2.89; 95%CI: 1.08–7.69, p = 0.034) and Kintampo (OR = 2.59; 95%CI: 1.66–4.04, p<0.001), exposure to either larval or adult stages of *T. solium* in Ifakara (OR = 15.42; 95%CI: 1.84–129.10, p = 0.012), exposure to *T. canis* in all study site (OR = 1.52; 95%CI: 1.23–1.87, p<0.001), Ifakara (OR = 2.15; 95%CI: 1.46–3.18, p<0.001) and Kilifi (OR = 1.64; 95%CI: 1.11–2.40, p = 0.012), *T. gondii* in all study sites (OR = 1.28; 95%CI: 1.04–1.56, p = 0.018) and exposure to HIV in Kilifi (OR = 3.00; 95%CI: 1.12–8.04,p = 0.028), all of which were associated with increased prevalence of ACE [10]. The association between seropositivity and ACE on analysis of HIV negative individuals (exclusion of 182 controls and 140 cases who were HIV positive) had similar results with the exception of *T. gondii*, in which the positive association was not statistically significant (Table S6).

### Magnitude of antibody response and prevalence of ACE

**Figure 1:**
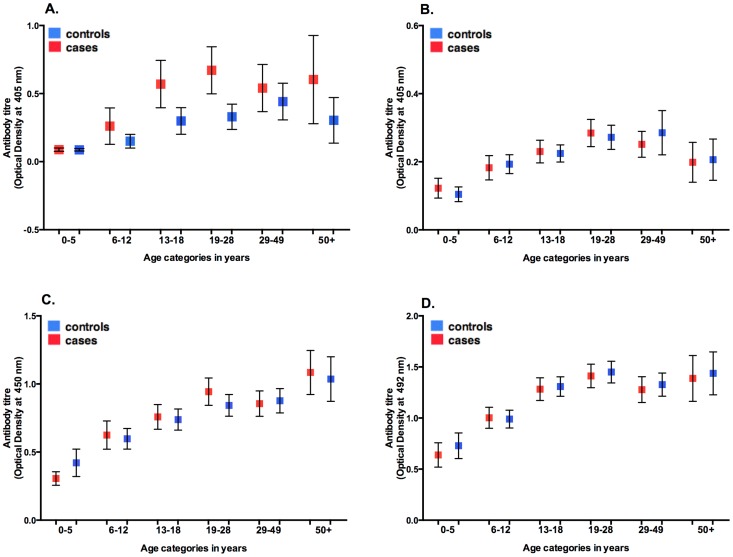
Antibody levels to *Onchocerca volvulus, Toxocara canis, Toxoplasma gondii* and *Plasmodium falciparum.* Mean antibody levels in cases and controls and by age category in A. *Onchocerca volvulus* B. *Toxocara canis* C. *Toxoplasma gondii* D. *Plasmodium falciparum*. Bars indicate 95% confidence intervals. Pooled data from all study sites.

**Figure 2:**
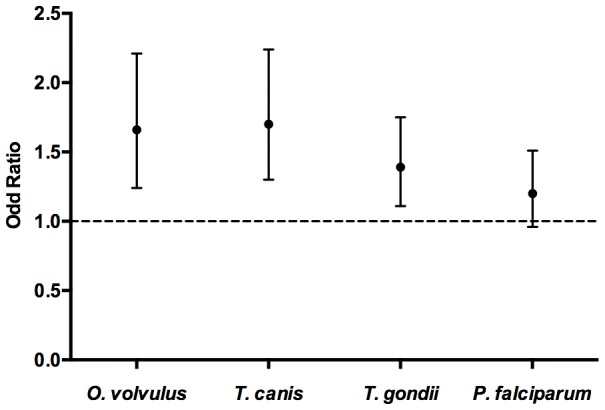
Association between ACE and high antibody levels to *O. volvulus, T. canis, T. gondii* and *P. falciparum*. Association between the top antibody tertile to *Onchocerca volvulus*, *Toxocara canis*, *Toxoplasma gondii* and *Plasmodium falciparum* and prevalence of ACE using pooled data from the five study sites. Age, sex, study-site, education, employment and marital status adjusted odds ratios. Dotted line represents an odds ratio of one and bars indicate 95% confidence intervals.

There was an increase in antibody levels with age in both cases and controls for *O. volvulus*, *T. canis*, *T. gondii* and *P. falciparum* (Figure 1) with the trend consistent in individual study sites. ACE was associated with high antibody levels (top tertile) to *O. volvulus*, *T. canis* and *T. gondii*, although the associations varied in magnitude across the study sites (Figure 2 and Table 2, S2–S5). Exposure to *P. falciparum* alone was not significantly associated with increased risk of ACE in any of the study sites.

**Table 2:** Association between ACE and antibody levels to *Onchocerca volvulus, Toxocara canis, Toxoplasma gondii* and *Plasmodium falciparum* across all study sites.

Parasitic infection	Antibody Tertiles	Univariate analysis		Multivariate analysis#	
		OR(95% CI) **	P-value	OR(95% CI) **	P-value
*Onchocerca Volvulus* *	Mid Tertile	1.02(0.78–1.34)	0.876	0.99(0.74–1.32)	0.962
	Top Tertile	**1.63(1.26–2.13)**	**<0.001**	**1.66(1.24–2.21)**	**0.001**
*Toxocara canis*	Mid Tertile	**1.32(1.02–1.70)**	**0.032**	**1.34(1.02–1.75)**	**0.036**
	Top Tertile	**1.68(1.30–2.16)**	**0.001**	**1.70(1.30**–**2.24)**	**<0.001**
*Toxoplasma gondii*	Mid Tertile	**1.42(1.15–1.74)**	**0.001**	**1.35(1.08–1.68)**	**0.008**
	Top Tertile	**1.42(1.16–1.75)**	**0.001**	**1.39(1.11–1.75)**	**0.004**
*Plasmodium falciparum*	Mid Tertile	0.99(0.81–1.22)	0.968	0.90(0.72–1.13)	0.366
	Top Tertile	**1.27(1.04–1.56)**	**0.020**	1.20(0.96–1.51)	0.114

# Logistic regression model included age, sex, study site, education (none, primary, or secondary and above), employment and marital status.

*Tested in 3 endemic sites: Ifakara, Iganga and Kintampo.

** Odds ratio compares mid and top tertile with lowest tertile

As shown in Table 2 and Figure 2 using pooled data from all study sites, high antibody levels (top tertile) were significantly associated with increased prevalence of ACE for *O. volvulus* (OR = 1.66; 95%CI: 1.24–2.21, p = 0.001), *T. canis* (OR = 1.70; 95%CI: 1.30–2.24, p<0.001) and *T. gondii* (OR = 1.39; 95%CI: 1.11–1.75, p = 0.004). A dose-response association was observed for *Toxocara canis* infection both in the pooled analysis (Table 2) and in analysis of individual sites, with the exception of Iganga-Mayuge (Table S3). The results were similar in an analysis of HIV negative individuals (Table S7).

### Exposure to multiple infections and prevalence of ACE

73.8% of cases and 65.5% of controls that were tested for exposure to all six infections had evidence of exposure to two or more infections (data not shown). A high proportion of individuals were exposed to both *T. gondii* and *P. falciparum* (35%), both *O. volvulus* and *P. falciparum* (28%), and *T. canis* and *P. falciparum* (23%). The remaining combinations occurred in less than 15% of individuals.

With the exception of exposure to *T. solium* and HIV (OR = 0.82; 95%CI: 0.15–4.45, p = 0.818), exposure to multiple infectious agents was associated with an increased prevalence of ACE after adjusting for age, sex, study site, education (none, primary, or secondary and above), employment, marital status and exposure to other assayed infections with the majority remaining statistically significant (Table 3). The results were similar in an analysis of HIV negative individuals (Table S8).

**Table 3:** Association between exposure to multiple infections and prevalence of ACE.

Exposure to multiple infections	Control	Cases	Unadjusted analysis		Adjusted analysis#	
	N(%)	N(%)	OR(95% CI)	P-value	OR(95% CI)	P-value
*Toxocara canis + Toxoplasma gondii*	116(9.0)	147(15.0)	1.91(1.45–2.50)	**<0.001**	1.65(1.11–2.45)	**0.014**
*Toxocara canis + Onchocerca volvulus*	84(11.0)	104(20.0)	2.55(1.84–3.53)	**<0.001**	2.34(1.62–3.37)	**<0.001**
*Toxocara canis +Taenia solium*	5(0.6)	6(1.2)	2.17(0.67–7.18)	0.202	1.65(0.46–5.86)	0.440
*Toxocara canis* + HIV	32(2.5)	30(3.2)	1.41(0.85–2.35)	0.183	1.04(0.56–1.95)	0.896
*Toxocara canis + Plasmodium falciparum*	254(20.0)	269(28.0)	1.40(1.07–1.84)	**0.015**	3.63(1.14–11.47)	**0.028**
*Toxoplasma gondii + Onchocerca volvulus*	100(12.0)	135(26.0)	2.68(1.96–3.66)	**<0.001**	2.53(1.72–3.75)	**0.001**
*Toxoplasma gondii + Taenia solium*	7(0.9)	8(1.6)	2.12(0.76–5.91)	0.152	1.73(0.57–5.18)	0.325
*Toxoplasma gondii* + HIV	78(6.0)	69(7.0)	1.26(0.89–1.77)	0.194	1.35(0.86–2.11)	0.192
*Toxoplasma gondii + Plasmodium falciparum*	435(34.0)	362(37.0)	1.04(0.81–1.33)	0.771	11.66(1.48–91.53)	**0.020**
*Onchocerca volvulus + Taenia solium*	5(0.6)	9(1.7)	3.51(1.17–10.55)	**0.026**	3.23(1.02–10.16)	**0.045**
*Onchocerca volvulus +* HIV	32(3.8)	33(6.2)	2.07(1.25–3.44)	**0.005**	1.74(0.94–3.18)	0.075
*Onchocerca volvulus + Plasmodium falciparum*	186(22.0)	202(38.0)	2.58(1.10–6.03)	**0.029**	3.81(1.29–11.17)	**0.015**
*Taenia solium +* HIV	5(0.6)	2(0.4)	0.65(0.13–3.38)	0.611	0.82(0.15–4.45)	0.818
*Taenia solium + Plasmodium falciparum*	18(2.2)	15(2.8)	2.50(0.84–7.48)	0.101	3.13(0.87–11.23)	0.080
HIV *+ Plasmodium falciparum*	144(11.0)	111(11.0)	0.98(0.70–1.36)	0.889	2.97(0.92–9.57)	0.068

# Logistic regression model included age, sex, study site, education (none, primary, or secondary and above), employment, marital status and exposure to other assayed infections. The odds ratio is comparing odds of being a case in co-infected versus uninfected.

### Interaction between exposures to multiple parasites

There was evidence of interaction on an additive scale in individuals exposed to *T. gondii* and *O. volvulus* (RERI = 1.19; 95%CI: 0.27–2.11, p = 0.011) (Table 4), which implies that the combined effect of these parasites is greater than the sum of the individual effects. Interaction could not be determined for *O. volvulus* and *P. falciparum* co-infection as all cases of ACE with exposure to *O. volvulus* were also exposed to *P. falciparum*. The results were similar in an analysis of HIV negative individuals (Table S9).

**Table 4:** Interaction on an additive scale between the effects of parasites on the prevalence of ACE.

Exposure to multiple infections	Relative excess risk due to interaction (RERI)	P-value
*Toxocara canis+Toxoplasma gondii*	0.63(−0.03–1.29)	0.063
*Toxocara canis+Onchocerca volvulus*	−0.16(−1.28–0.97)	0.785
*Toxocara canis+Plasmodium falciparum*	−3.35(−18.27–11.58)	0.660
*Toxoplasma gondii+Onchocerca volvulus*	1.19(0.27–2.11)	**0.011**
*Toxoplasma gondii+Plasmodium falciparum*	−18.24(−68.93–32.46)	0.481
*Onchocerca volvulus+Taenia solium*	1.33(−2.48–5.79)	0.481
*Onchocerca volvulus+Plasmodium falciparum*	n.d*	n.d

^*^Interaction could not be determined for *O. volvulus* and *P. falciparum* co-infection as there were no cases of ACE with exposure to *O. volvulus* infection without exposure to *P. falciparum*.

+RERI adjusted for age, sex, study site, education (none, primary, or secondary and above), employment, marital status and exposure to other assayed infections. A positive RERI indicates that the combined effect of the two parasites is greater than the sum of the individual effects.

## Discussion

People living in SSA are exposed to multiple parasites, some of which are associated with epilepsy[5], [7]. We have shown that exposure to individual parasites (*O. volvulus, T. canis* and *T. gondii*) is associated with an increased prevalence of ACE, and for *T. canis* there is a dose response relationship between antibody level and ACE. For co-infection with *T. gondii* and *O. volvulus*, the combined effect was greater than the sum of the individual effects.

Exposure to *O. volvulus, T. canis* and *T. gondii* was associated with an increased prevalence of epilepsy in other case-control studies [8], [9], [10], [11], [12], [13], [28], [29], [30]. Previous studies reporting the association between parasitic infection and epilepsy have used seropositivity to define exposure[8], [9], [14], [28], [29], [31], [32], [33], with none taking into account the degree of exposure as a predictor for risk of ACE. Elevated antibody responses could reflect recent or current infection or could serve as a proxy for estimating the level or degree of exposure as antibody levels would be elevated due to repeated infections. This may explain previously conflicting results, such as the increased prevalence of epilepsy with exposure to *T. canis* in Burundi[9] and the absence of an association with epilepsy in Tanzania[8]. Furthermore, even within an area, there is heterogeneity in the prevalence of epilepsy[6], which may be explained by differences in the levels of exposure to certain parasites.

With the exception of cysticercosis, the epileptogenesis of parasitic infections is not entirely elucidated[5]. Neurocysticercosis is a well-known risk factor for epilepsy in South America and has also been identified as a risk factor in few studies in Africa [8], [14], [34], [35], [36]. In this study, the prevalence of antibodies to *T. solium* was low in all three-study sites. In Ifakara Tanzania, the main agricultural activities include subsistence farming of maize, rice and cassava and fishing is their main source of protein intake and income[37]. The main inhabitants of Ifakara are both Christians and Muslims [37] and it is likely that pork consumption is low among the Muslim inhabitants. Similarly, fishing and farming are the main agricultural activities in Kintampo (http://www.indepth-network.org) and the main inhabitants include a large migrant population from the north who are mainly Muslim[38]. The absence of an association in this study is likely to be attributed to cultural practices in which free-range pig rearing and pork consumption is not common [37], [38]. In addition, the low sensitivity of the detection assay for identifying single viable cysts, calcified cysts or degenerating cysts [20] that could be epileptogenic[39] and possibly the lack of the statistical power to detect an association due to a low prevalence of *T. solium* antibodies. Other studies have found that a history of admission with cerebral malaria or malaria complicated with seizures have been previously shown to be associated with ACE [10], [15]. The lack of association between *P. falciparum* serology and ACE in our study might be because serology cannot currently distinguish between asymptomatic exposure, mild malaria, malaria complicated with seizure or cerebral malaria.

We show that exposure to multiple parasitic infections as well as HIV was relatively common and was strongly associated with an increased prevalence of ACE. Although few studies have analysed exposure to two parasites such as cysticercosis and toxocariasis[8] or three parasites, cysticercosis, toxocariasis and paragonimiasis[33], these studies did not report on the risk of epilepsy associated with exposure to multiple infections. We provide evidence of interaction for an additive model of *O. volvulus* and *T. gondii* co-infection.

The evidence of interaction in individuals exposed to *T. gondii* and *O. volvulus* may be explained by the predominant immune responses induced by the different infections. Interferon gamma (IFNγ), a Th1 response has been shown to be essential for controlling *T. gondii* infection[40]. Th2 responses dominate in chronic filarial infections such as *Brugia malayi* (in a murine model)[41] and *O. volvulus* [42] and this tends to suppress Th1 responses. These observations suggest that individuals with chronic *O. volvulus* infection who are co-infected with *T. gondii* may be unable to mount an adequate protective response resulting in increased severity of disease e.g. due to the rupture of cysts containing *T. gondii* that are then epileptogenic[43], [44]. There is a need for additional studies in both humans and in animal models to elucidate the mechanisms of pathogenesis with these parasitic infections[5] as well as in models of co-infections in order to gain insight to their role in epilepsy.

We measured exposure to infections using well-established and robust techniques. IgG antibodies to *Toxocara* excretory secretory antigen remain elevated for many years in chronic infections and have been shown to persist for over 4 years after curative treatment[45]. IgG antibodies to *T. gondii* purified antigens are detectable months after infection[46]. Exposure to *P. falciparum* determined by detection of IgG antibodies to schizont extract was selected as these antibodies are known to be long-lived and detectable up to 11 years in the absence of antigenic re-stimulation[47]. With the exception of *T. solium* [20] and *O. volvulus* whose antibody longevity is unknown, our measurement would detect a current or prior exposure to infection.

We used a standardised approach to determine exposure to infections across all study sites, this exposure was determined after the onset of seizures, and as such, it is difficult to confirm a causal link. Despite adjusting for socio-economic status in our statistical analysis, this may not have adequately measured poverty and there might be residual confounding. In addition, the required sample size was not achieved and may explain the differences between the expected and observed associations. The findings from this study should be validated using longitudinal studies that monitor exposure to infections, which may help establish a causal link between parasitic infections and epilepsy. While it is not clear whether it is the presence of the parasite in the CNS or the immunological response to the infection that is epileptogenic, efforts to control these infections are likely to reduce the burden of epilepsy in SSA and should be explored using randomized intervention studies.

Control is possible with ivermectin for individual and mass-treatment of onchocerciasis[48],[49] niclosamide or praziquantel for treatment of taeniasis as well as albendazole or praziquantel for treatment of parasitic cysts such as in *T. solium* [50] and *T. canis* infection[51]. In addition, efforts to improve sanitation and personal hygiene practices, including safe food consumption practices, will reduce transmission of *T. canis*, *T. gondii* and *T. solium* infections. Safe pig rearing (i.e., separation from human waste contact) will further impact on *T. solium* transmission. Vector control measures as well as bed net usage, intermittent preventative treatment and effective chemotherapy are available for control *P. falciparum* infection[52]. These control measures should be explored and their contribution to the burden of ACE evaluated. While feasible control measures are known, their use depends largely on a wider commitment to improving public health.

We have shown that the intensity of exposure to certain infections and multiple parasitic infections is associated with increased prevalence of ACE and may explain conflicting results obtained when only seropositivity is considered. A recent study indicated that approximately 35% of ACE cases in adults in SSA are attributed to parasitic infections[10]. The findings from this study should be further validated using longitudinal studies to confirm a causal link between parasitic infection and epilepsy. Thereafter, randomized intervention studies targeting each parasitic infection should be explored and their contribution to the burden of ACE evaluated.

## Supporting Information

Checklist S1 **STROBE checklist for case-control studies.**
(DOC)

Figure S1 **Prevalence of antibodies to** ***Onchocerca volvulus*** **.** Prevalence of IgG antibodies to *Onchocerca volvulus* in A. Ifakara B. Iganga and C. Kintampo in cases and controls and by age category.
(TIFF)

Figure S2 **Prevalence of antibodies to** ***Toxocara canis*** **.** Prevalence of IgG4 antibodies to *Toxocara canis* in A. Agincourt B. Ifakara C. Iganga D. Kilifi and E. Kintampo in cases and controls and by age category.
(TIFF)

Figure S3 **Prevalence of antibodies to** ***Toxoplasma gondii*** **.** Prevalence of IgG antibodies to *Toxoplasma gondii* in A. Agincourt B. Ifakara C. Iganga D. Kilifi and E. Kintampo in cases and controls and by age category.
(TIFF)

Figure S4 **Prevalence of antibodies to** ***Plasmodium falciparum*** **.** Prevalence of IgG antibodies to *Plasmodium falciparum* in A. Agincourt B. Ifakara C. Iganga D. Kilifi and E. Kintampo in cases and controls and by age category.
(TIFF)

Figure S5 **Prevalence of antibodies to** ***Taenia solium*** **.** Prevalence of IgG antibodies to *Taenia solium* in A. Ifakara B. Iganga and C. Kintampo in cases and controls and by age category.
(TIFF)

Figure S6 **Prevalence of antibodies to HIV.** Prevalence of IgG antibodies to HIV in A. Agincourt B. Ifakara C. Iganga D. Kilifi and E. Kintampo in cases and controls and by age category.
(TIFF)

Table S1 **Demographic characteristics of cases and controls from each study site.**
(DOC)

Table S2 **Association between IgG4 antibody titers to** ***Onchocerca volvulus*** **and prevalence of ACE.**
(DOC)

Table S3 **Association between IgG4 antibody titers to** ***Toxocara canis*** **and prevalence of ACE.**
(DOC)

Table S4 **Association between IgG antibody titers to** ***Toxoplasma gondii*** **and prevalence of ACE.**
(DOC)

Table S5 **Association between IgG antibody titers to** ***Plasmodium falciparum*** **and prevalence of ACE.**
(DOC)

Table S6 **Table showing the seropositivity to six different infections and association with ACE in HIV negative individuals using pooled data from all study sites.**
(DOC)

Table S7 **Association between ACE and antibody levels to** ***Onchocerca volvulus, Toxocara canis, Toxoplasma gondii*** **and** ***Plasmodium falciparum*** **in HIV negative individuals across all study sites.**
(DOC)

Table S8 **Association between exposure to multiple infections and prevalence of ACE in HIV negative individuals across all study sites.**
(DOC)

Table S9 **Interaction on an additive scale between the effects of parasites on the prevalence of ACE in HIV negative individuals.**
(DOC)
