# Supplementary material for: Exposure to Multiple Parasites Is Associated with the Prevalence of Active Convulsive Epilepsy in Sub-Saharan Africa
Source: PLoS Negl Trop Dis. 2014 May 29;8(5):e2908. doi: 10.1371/journal.pntd.0002908 (PMC4038481; doi:10.1371/journal.pntd.0002908)
Supplement: Table S1 — Demographic characteristics of cases and controls from each study site. (DOC) [file pntd.0002908.s008.doc]

Supplementary information

Table S1: Demographic characteristics of cases and controls from each study site.

|  |  | All study sites | | | Agincourt, South Africa | | Ifakara, Tanzania | | Iganga-Mayuge, Uganda | | Kilifi, Kenya | | Kintampo, Ghana | |
| --- | --- | --- | --- | --- | --- | --- | --- | --- | --- | --- | --- | --- | --- | --- |
|  |  | Control  %  (N=1313 | Case  %  (986) | OR (95% CI)  P-value | Control %  (N=211) | Case  %  (N=175) | Control  %  (N=345) | Case  %  (N=278) | Control %  (N=199) | Case  %  (N=84) | Control %  (N=266) | Case  %  (N=276) | Control %  (N=292) | Case  %  (N=173) |
| Age categories in years | 0-5 | 7 | 7 | - | 2 | 2 | 5 | 5 | 19 | 27 | 12 | 11 | 2 | 1 |
|  | 6-12 | 21 | 17 | 0.75 (0.59-0.96)  **0.023** | 18 | 8 | 26 | 22 | 30 | 25 | 18 | 20 | 16 | 12 |
|  | 13-18 | 21 | 22 | 0.93 (0.72-1.18)  0.557 | 10 | 15 | 22 | 23 | 18 | 17 | 26 | 25 | 27 | 25 |
|  | 19-28 | 22 | 23 | 1.04 (0.82-1.33)  0.721 | 21 | 22 | 17 | 19 | 20 | 15 | 22 | 21 | 32 | 37 |
|  | 29-49 | 20 | 22 | 0.89 (0.70-1.15)  0.387 | 34 | 34 | 24 | 26 | 11 | 12 | 14 | 14 | 18 | 19 |
|  | 50+ | 7 | 9 | 0.91 (0.67-1.23)  0.551 | 15 | 19 | 7 | 5 | 3 | 4 | 8 | 10 | 5 | 6 |
| Sex | Female | 54 | 49 | - | 32 | 55 | 51 | 48 | 42 | 52 | 49 | 48 | 48 | 55 |
|  | Male | 46 | 51 | 1.2 (1.05-1.38)  **0.003** | 68 | 45 | 49 | 52 | 58 | 48 | 51 | 52 | 52 | 45 |
| Married or mothers marital status | No | 32 | 48 | - | 55 | 71 | 28 | 40 | 18 | 30 | 28 | 42 | 33 | 56 |
|  | Yes | 68 | 52 | 0.53 (0.47-0.61)  **<0.001** | 45 | 29 | 72 | 60 | 82 | 70 | 72 | 58 | 67 | 44 |
| Earns cash or mother/father earn cash | No | 27 | 41 | - | 72 | 85 | 7 | 14 | 27 | 46 | 29 | 41 | 17 | 35 |
|  | Yes | 73 | 59 | 0.52 (0.47-0.59)  **<0.001** | 28 | 15 | 93 | 86 | 73 | 54 | 71 | 59 | 83 | 65 |
| Education level of interviewee | None | 30 | 38 | - | 22 | 23 | 15 | 31 | 22 | 35 | 45 | 51 | 43 | 45 |
|  | Primary | 38 | 39 | 0.81 (0.69-0.93)  **0.005** | 35 | 39 | 59 | 54 | 43 | 48 | 45 | 41 | 6 | 5 |
|  | Secondary and above | 24 | 17 | 0.68 (0.56-0.82)  **<0.001** | 43 | 35 | 3 | 1 | 25 | 13 | 7 | 4 | 49 | 47 |
|  | Unknown | 9 | 7 | 0.58 (0.45-0.75)  **<0.001** | 1 | 3 | 23 | 14 | 10 | 5 | 3 | 4 | 2 | 4 |
