# Supplementary material for: Exposure to Multiple Parasites Is Associated with the Prevalence of Active Convulsive Epilepsy in Sub-Saharan Africa
Source: PLoS Negl Trop Dis. 2014 May 29;8(5):e2908. doi: 10.1371/journal.pntd.0002908 (PMC4038481; doi:10.1371/journal.pntd.0002908)
Supplement: Table S2 — Association between IgG4 antibody titers to Onchocerca volvulus and prevalence of ACE. (DOC) [file pntd.0002908.s009.doc]

Table S2: Association between IgG4 antibody titers to *Onchocerca volvulus* and prevalence of ACE.

| Study site | Antibody Tertiles | Univariate analysis | | Multivariate analysis^#^ | |
| --- | --- | --- | --- | --- | --- |
|  |  | OR (95 % CI) * | P-value | OR (95 % CI) * | P-value |
| Ifakara | Mid Tertile | 0.76 (0.52-1.13) | 0.177 | 0.79 (0.53-1.20) | 0.280 |
|  | Top Tertile | 1.38 (0.94-2.03) | 0.098 | 1.35 (0.89-2.06) | 0.158 |
| Iganga | Mid Tertile | 0.59 (0.31-1.10) | 0.101 | **0.46 (0.23-0.94)** | **0.034** |
|  | Top Tertile | 0.63 (0.34-1.16) | 0.143 | 0.62 (0.32-1.24) | 0.181 |
| Kintampo | Mid Tertile | **2.23 (1.35-3.67)** | **0.002** | **2.29 (1.32-3.94)** | **0.003** |
|  | Top Tertile | **3.82 (2.34-6.27)** | **<0.001** | **4.05 (2.34-6.99)** | **<0.001** |

^#^Logistic regression model included age, sex, education (none, primary, or secondary and above), employment and marital status. ***** OR compares mid and top tertile with lowest tertile.
