# Supplementary material for: Exposure to Multiple Parasites Is Associated with the Prevalence of Active Convulsive Epilepsy in Sub-Saharan Africa
Source: PLoS Negl Trop Dis. 2014 May 29;8(5):e2908. doi: 10.1371/journal.pntd.0002908 (PMC4038481; doi:10.1371/journal.pntd.0002908)
Supplement: Table S3 — Association between IgG4 antibody titers to Toxocara canis and prevalence of ACE. (DOC) [file pntd.0002908.s010.doc]

Table S3: Association between IgG4 antibody titers to *Toxocara canis* and prevalence of ACE.

| Study site | Antibody Tertile | Univariate Analysis | | Multivariate analysis^#^ | |
| --- | --- | --- | --- | --- | --- |
|  |  | OR (95% CI) * | P-value | OR (95% CI) * | P-value |
| Agincourt | Mid Tertile | 0.83 (0.41-1.66) | 0.591 | 1.17 (0.51-2.67) | 0.711 |
|  | Top Tertile | 1.02 (0.52-2.01) | 0.945 | 1.44 (0.65-3.20) | 0.370 |
| Ifakara | Mid Tertile | **1.96 (1.12-3.42)** | **0.017** | **2.02 (1.11-3.65)** | **0.020** |
|  | Top Tertile | **2.51 (1.44-4.40)** | **0.001** | **2.72 (1.46-5.06)** | **0.002** |
| Iganga | Mid Tertile | 1.52 (0.69-3.34) | 0.295 | 1.58 (0.66-3.78) | 0.309 |
|  | Top Tertile | 1.28 (0.58-2.81) | 0.540 | 1.17 (0.46-2.95) | 0.735 |
| Kilifi | Mid Tertile | 1.37 (0.85-2.19) | 0.189 | 1.35 (0.81-2.24) | 0.247 |
|  | Top Tertile | **1.89 (1.17-3.06)** | **0.009** | **1.73 (1.02-2.93)** | **0.040** |
| Kintampo | Mid Tertile | 1.10 (0.65-1.87) | 0.722 | 1.14 (0.63-2.06) | 0.659 |
|  | Top Tertile | 1.65 (0.98-2.78) | 0.059 | 1.85 (1.05-3.28) | **0.034** |

^#^Logistic regression model included age, sex, education (none, primary, or secondary and above), employment and marital status. ***** OR compares mid and top tertile with lowest tertile.
