# Supplementary material for: Exposure to Multiple Parasites Is Associated with the Prevalence of Active Convulsive Epilepsy in Sub-Saharan Africa
Source: PLoS Negl Trop Dis. 2014 May 29;8(5):e2908. doi: 10.1371/journal.pntd.0002908 (PMC4038481; doi:10.1371/journal.pntd.0002908)
Supplement: Table S4 — Association between IgG antibody titers to Toxoplasma gondii and prevalence of ACE. (DOC) [file pntd.0002908.s011.doc]

Table S4: Association between IgG antibody titers to *Toxoplasma gondii* and prevalence of ACE.

| Study site | Antibody Tertile | Univariate Analysis | | Multivariate analysis^#^ | |
| --- | --- | --- | --- | --- | --- |
|  |  | OR (95% CI) * | P-value | OR (95% CI) * | P-value |
| Agincourt | Mid Tertile | **1.74 (1.06-2.87)** | **0.028** | 1.61 (0.93-2.78) | 0.085 |
|  | Top Tertile | **1.72 (1.04-2.83)** | **0.034** | 1.64 (0.93-2.89) | 0.090 |
| Ifakara | Mid Tertile | 1.09 (0.74-1.64) | 0.651 | 1.04 (0.67-1.59) | 0.872 |
|  | Top Tertile | 1.24 (0.83-1.85) | 0.288 | 1.17 (0.75-1.81) | 0.482 |
| Iganga | Mid Tertile | 1.10 (0.59-2.05) | 0.763 | 1.17 (0.59-2.35) | 0.646 |
|  | Top Tertile | 0.98 (0.52-1.83) | 0.946 | 1.13 (0.54-2.36) | 0.743 |
| Kilifi | Mid Tertile | 1.46 (0.96-2.20) | 0.074 | 1.46 (0.93-2.30) | 0.104 |
|  | Top Tertile | 1.39 (0.92-2.10) | 0.116 | **1.64 (1.02-2.66)** | **0.043** |
| Kintampo | Mid Tertile | **1.92 (1.19-3.08)** | **0.007** | **1.70 (1.01-2.87)** | **0.046** |
|  | Top Tertile | **1.89 (1.18-3.05)** | **0.008** | **1.71 (1.00-2.92)** | **0.050** |

^#^Logistic regression model included age, sex, education (none, primary, or secondary and above), employment and marital status. ***** OR compares mid and top tertile with lowest tertile.
