# Supplementary material for: Exposure to Multiple Parasites Is Associated with the Prevalence of Active Convulsive Epilepsy in Sub-Saharan Africa
Source: PLoS Negl Trop Dis. 2014 May 29;8(5):e2908. doi: 10.1371/journal.pntd.0002908 (PMC4038481; doi:10.1371/journal.pntd.0002908)
Supplement: Table S5 — Association between IgG antibody titers to Plasmodium falciparum and prevalence of ACE. (DOC) [file pntd.0002908.s012.doc]

Table S5: Association between IgG antibody titers to *Plasmodium falciparum* and prevalence of ACE.

| Study site | Antibody Tertile | Univariate Analysis | | Multivariate analysis^#^ | |
| --- | --- | --- | --- | --- | --- |
|  |  | OR (95% CI) * | P-value | OR (95% CI) * | P-value |
| Agincourt | Mid Tertile | 0.83 (0.53-1.44) | 0.598 | 0.92 (0.53-1.59) | 0.760 |
|  | Top Tertile | 1.50 (0.91-2.45) | 0.107 | 1.44 (0.83-2.49) | 0.196 |
| Ifakara | Mid Tertile | 1.19 (0.81-1.76) | 0.372 | 1.12 (0.73-1.68) | 0.630 |
|  | Top Tertile | 1.41 (0.95-2.07) | 0.085 | 1.39 (0.91-2.13) | 0.132 |
| Iganga | Mid Tertile | 0.97 (0.52-1.80) | 0.912 | 0.93 (0.45-1.90) | 0.839 |
|  | Top Tertile | 0.92 (0.49-1.71) | 0.782 | 1.09 (0.51-2.36) | 0.814 |
| Kilifi | Mid Tertile | 0.84 (0.55-1.27) | 0.400 | 0.71 (0.44-1.16) | 0.171 |
|  | Top Tertile | 1.38 (0.91-2.10) | 0.125 | 1.39 (0.83-2.32) | 0.214 |
| Kintampo | Mid Tertile | 1.09 (0.68-1.72) | 0.724 | 0.98 (0.59-1.61) | 0.929 |
|  | Top Tertile | 1.06 (0.67-1.68) | 0.814 | 0.89 (0.53-1.50) | 0.683 |

^#^Logistic regression model included age, sex, education (none, primary, or secondary and above), employment and marital status. ***** OR compares mid and top tertile with lowest tertile.
