# Supplementary material for: Exposure to Multiple Parasites Is Associated with the Prevalence of Active Convulsive Epilepsy in Sub-Saharan Africa
Source: PLoS Negl Trop Dis. 2014 May 29;8(5):e2908. doi: 10.1371/journal.pntd.0002908 (PMC4038481; doi:10.1371/journal.pntd.0002908)
Supplement: Table S6 — Table showing the seropositivity to six different infections and association with ACE in HIV negative individuals using pooled data from all study sites. (DOC) [file pntd.0002908.s013.doc]

Table S6: Table showing the seropositivity to six different infections and association with ACE in HIV negative individuals using pooled data from all study sites.

|  | All study sites | | |
| --- | --- | --- | --- |
|  | Control % | Case  % | OR*  (95% CI)  P-value |
| **No of Individuals** | **1131** | **846** |  |
| *Plasmodium falciparum* + | 83.4 | 82.2 | 1.15  (0.84-1.57)  0.391 |
| *Toxocara canis* + | 23.0 | 32.7 | **1.56**  **(1.25-1.95)**  **<0.001** |
| *Toxoplasma gondii* + | 34.1 | 37.42 | 1.22  (0.98-1.52)  0.072 |
| *Onchocerca volvulus +* | 22.1 | 38.1 | **2.05**  **(1.53-2.74)**  **<0.001** |
| Cysticercosis + | 0.9 | 2.1 | 2.23  (0.76-6.49)  0.143 |
| Taeniasis + | 1.1 | 1.1 | 1.22  (0.36-4.10)  0.745 |
| Cysticercosis and Taeniasis+ | 1.8 | 3.0 | 1.79  (0.78-4.10)  0.167 |

*Odds ratio (OR) adjusted for age, sex, study site, education (none, primary, or secondary and above), employment and marital status.
