# Supplementary material for: Exposure to Multiple Parasites Is Associated with the Prevalence of Active Convulsive Epilepsy in Sub-Saharan Africa
Source: PLoS Negl Trop Dis. 2014 May 29;8(5):e2908. doi: 10.1371/journal.pntd.0002908 (PMC4038481; doi:10.1371/journal.pntd.0002908)
Supplement: Table S7 — Association between ACE and antibody levels to Onchocerca volvulus, Toxocara canis, Toxoplasma gondii and Plasmodium falciparum in HIV negative individuals across all study sites. (DOC) [file pntd.0002908.s014.doc]

Table S7: Association between ACE and antibody levels to *Onchocerca volvulus, Toxocara canis, Toxoplasma gondii* and *Plasmodium falciparum* in HIV negative individuals across all study sites

^#^Logistic regression model included age, sex, study site, education (none, primary, or secondary and above), employment and marital status. *Tested in 3 endemic sites: Ifakara, Iganga and Kintampo. ** OR compares mid and top tertile with lowest tertile

| Parasitic infection | Antibody Tertiles | Multivariate analysis^#^ | |
| --- | --- | --- | --- |
|  |  | OR (95% CI) ** | P-value |
| *Onchocerca*  *Volvulus ** | Mid Tertile | 0.90 (0.66-1.24) | 0.527 |
|  | Top Tertile | **1.58 (1.16-2.17)** | **0.004** |
| *Toxocara*  *canis* | Mid Tertile | **1.41 (1.05-1.89)** | **0.022** |
|  | Top Tertile | **1.76 (1.31-2.37)** | **<0.001** |
| *Toxoplasma gondii* | Mid Tertile | 1.25 (0.98-1.58) | 0.069 |
|  | Top Tertile | **1.36 (1.06-1.74)** | **0.015** |
| *Plasmodium*  *falciparum* | Mid Tertile | 0.91 (0.72-1.16) | 0.454 |
|  | Top Tertile | **1.28 (1.00-1.64)** | **0.048** |
