# Supplementary material for: Exposure to Multiple Parasites Is Associated with the Prevalence of Active Convulsive Epilepsy in Sub-Saharan Africa
Source: PLoS Negl Trop Dis. 2014 May 29;8(5):e2908. doi: 10.1371/journal.pntd.0002908 (PMC4038481; doi:10.1371/journal.pntd.0002908)
Supplement: Table S8 — Association between exposure to multiple infections and prevalence of ACE in HIV negative individuals across all study sites. (DOC) [file pntd.0002908.s015.doc]

Table S8: Association between exposure to multiple infections and prevalence of ACE in HIV negative individuals across all study sites. ^#^Logistic regression model included age, sex, study site, education (none, primary, or secondary and above), employment, marital status and exposure to other assayed infections.

| Exposure to multiple infections | Control  N (%) | Cases  N (%) | Multivariate analysis^#^ | |
| --- | --- | --- | --- | --- |
|  | | | OR (95 % CI) | P-value |
| *Toxocara canis + Toxoplasma gondii* | 100 (9.2) | 127 (15.5) | 1.69 (1.10-2.60) | **0.017** |
| *Toxocara canis + Onchocerca volvulus* | 68 (10.1) | 86 (20.3) | 2.56 (1.70-3.85) | **<0.001** |
| *Toxocara canis +Taenia solium* | 3 (0.5) | 5 (1.2) | 1.86 (0.41-8.43) | 0.421 |
| *Toxocara canis + Plasmodium falciparum* | 226 (20.7) | 241 (29.2) | 4.21 (1.14-15.53) | **0.031** |
| *Toxoplasma gondii + Onchocerca volvulus* | 80 (11.6) | 108 (25.2) | 2.55 (1.67-3.92) | **<0.001** |
| *Toxoplasma gondii + Taenia solium* | 5 (0.7) | 6 (1.4) | 1.58 (0.43-5.81) | 0.490 |
| *Toxoplasma gondii + Plasmodium falciparum* | 359 (32.3) | 297 (35.7) | 9.13 (1.15-72.12) | **0.036** |
| *Onchocerca volvulus + Taenia solium* | 4(0.6) | 8(1.8) | 3.22 (0.91-11.35) | 0.069 |
| *Onchocerca volvulus + Plasmodium falciparum* | 154 (21.7) | 169 (38.1) | 3.85 (1.17-12.66) | **0.026** |
| *Taenia solium + Plasmodium falciparum* | 13(1.8) | 13(3.0) | 3.55 (0.85-14.86) | 0.082 |
