# Supplementary material for: Exposure to Multiple Parasites Is Associated with the Prevalence of Active Convulsive Epilepsy in Sub-Saharan Africa
Source: PLoS Negl Trop Dis. 2014 May 29;8(5):e2908. doi: 10.1371/journal.pntd.0002908 (PMC4038481; doi:10.1371/journal.pntd.0002908)
Supplement: Table S9 — Interaction on an additive scale between the effects of parasites on the prevalence of ACE in HIV negative individuals. (DOC) [file pntd.0002908.s016.doc]

Table S9: Interaction on an additive scale between the effects of parasites on the prevalence of ACE in HIV negative individuals.

| Exposure to multiple infections | Relative excess risk due to interaction (RERI)^+^ | P-value |
| --- | --- | --- |
| *Toxocara canis + Toxoplasma gondii* | 0.56 (-0.20-1.31) | 0.148 |
| *Toxocara canis + Onchocerca volvulus* | -0.25 (-1.57-1.08) | 0.718 |
| *Toxocara canis + Plasmodium falciparum* | -4.13 (-22.62-14.36) | 0.662 |
| *Toxoplasma gondii + Onchocerca volvulus* | 1.19 (0.18-2.21) | **0.021** |
| *Toxoplasma gondii + Plasmodium falciparum* | -15.26 (-61.16-30.63) | 0.515 |
| *Onchocerca volvulus + Plasmodium falciparum* | n.d | n.d |

n.d Interaction could not be determined for *O. volvulus* and *P. falciparum* co-infection as there were no cases of ACE with exposure to either *O. volvulus* infection without exposure to *P. falciparum.*

^+^ RERI adjusted for age, sex, study site, education (none, primary, or secondary and above), employment, marital status and exposure to other assayed infections. A positive RERI indicates that the combined effect of the two parasites is greater than the sum of the individual effects.
